# Idiopathic tumoral calcinosis of the left thigh: a case report

**DOI:** 10.1093/jscr/rjaf171

**Published:** 2025-03-28

**Authors:** Sami Eldirdiri, Samaual Eldirdiri, Ibrahim B A Elbashir, Nahid F Aldaw, Hind M A Abd Allah, Mohamed Y Ibrahim

**Affiliations:** Department of Surgery, University of Gadarif, Gadarif, Sudan; Department of Surgery, University of Gadarif, Gadarif, Sudan; Department of Orthopedic Surgery, University of Gadarif, Gadarif, Sudan; Head Department of Pathology, University of Gadarif, Gadarif, Sudan; Department of Oncology, University of Gadarif, Gadarif, Sudan; Pediatric Surgery Center, National Ribat University Hospital, Khartoum, Sudan

**Keywords:** idiopathic tumoral calcinosis, Sudan, case report, benign soft tissue lesion, “chicken wire” calcification

## Abstract

Idiopathic tumoral calcinosis is a very rare benign condition characterized by pathological deposition of calcium phosphate crystals into the soft tissues, particularly into the peri-articular regions. This case report narrates the first reported case of idiopathic tumoral calcinosis in Sudan. A 38-year-old Sudanese female from Gadarif presented with a history of progressive enlargement of the left thigh swelling over a period of 1 year, which was painless. Clinical examination revealed a firm and well-circumscribed mass without overlying skin changes. Imaging revealed calcification with a characteristic “chicken wire” pattern in X-rays, and histopathology confirmed lobulated calcium deposits surrounded by fibrous tissue and foreign body giant cell reactions without malignancy. The patient underwent complete surgical excision of the mass under spinal anesthesia, and her recovery was uneventful. This case highlights the need for recognition of idiopathic tumoral calcinosis in the differential diagnosis of soft tissue masses, especially in the region where these are underreported.

## Introduction

Tumoral calcinosis is a very rare benign condition associated with pathological deposition of calcium phosphate crystals in soft tissues of the periarticular region. First reported in 1899, tumoral calcinosis may be distinguished as idiopathic or primary forms unrelated to systemic metabolic abnormalities and secondary forms related to chronic renal failure or hyperparathyroidism [1–3].

Idiopathic tumoral calcinosis generally involves Africans and people of the Middle East, mainly in childhood and early adulthood. Large joints such as the shoulders, hips, and elbows are common sites, while a few cases present in the thighs [4–6]. A “chicken wire” pattern is the classic radiographic presentation on X-ray that may help in distinguishing this lesion from other calcified lesions [7]. Despite improvements in various imaging modalities, diagnosis and management of idiopathic tumoral calcinosis remain challenging [8].

Although cases of idiopathic tumoral calcinosis have been reported from all over the world, including India [9], the Philippines [5], and Korea [8], to the best of our knowledge, this is the first reported case of idiopathic tumoral calcinosis from Sudan. The case herein reports a peculiar presentation of the left thigh mass and documents the clinical, radiological, and pathological findings to create awareness and understanding of this rare disease in a part of the world where this has not been described [1, 2].

## Case report

A 38-year-old Sudanese female from Gadarif, Eastern Sudan, without significant past medical history presented to surgical clinic with 1 year history of upper thigh and hip swelling. Initially, the patient sought medical advice for the swelling but subsequently neglected the recommendations. Over time, the mass progressively increased in size, prompting her to seek medical attention again after experiencing mild discomfort at the site.

On examination, a firm, non-tender, well-circumscribed mass measuring ⁓16 × 12 cm in diameter was palpated in the upper thigh, near the hip. The overlying skin appeared normal, without erythema or warmth. The mass hard in consistency and attached to deep structure but not to skin with positive inguinal lymph node, and limited range of movement was noticed.

**Figure 1:**
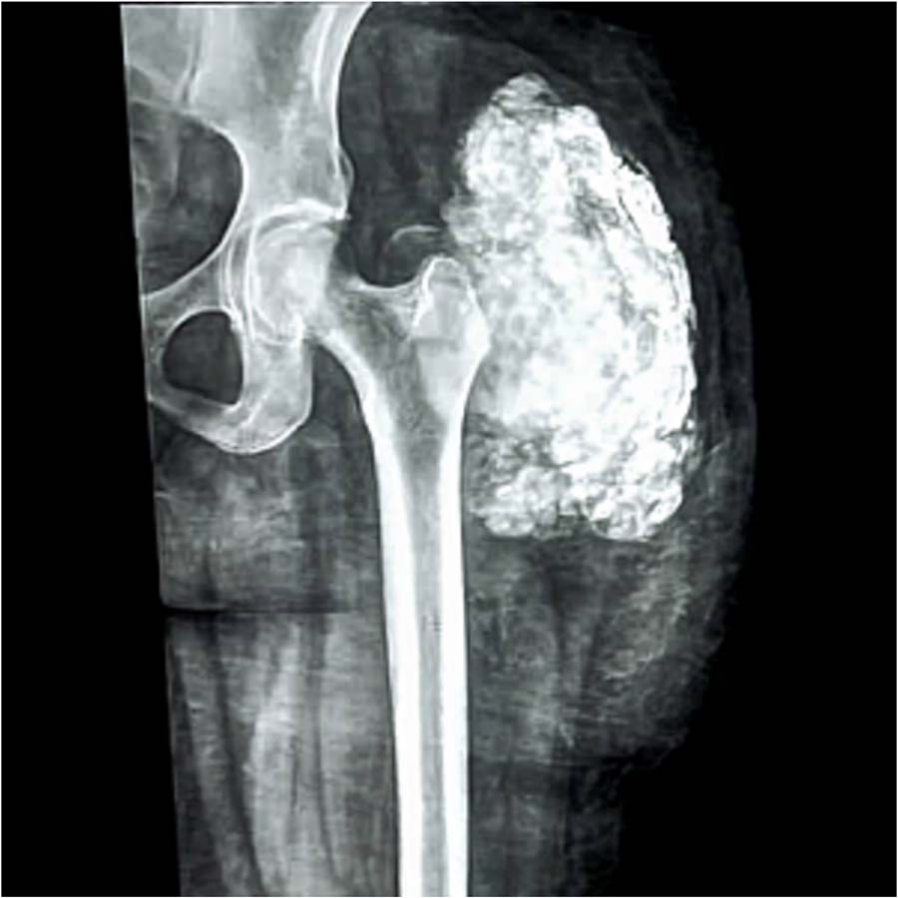
The X-ray of the left upper thigh and hip shows a calcified mass with a well-defined margin (“chicken wire” pattern calcification).

Blood tests: Complete blood count, viral screening, and renal function tests, including S. Phosphorus and S. Calcium, were all within normal range. Radiologically: The X-ray of the left upper thigh and hip shows a “cloud-like” calcified mass with a well-defined margin seen abutting the hip joint (Fig. 1). This was associated with typical “chicken wire” pattern calcification surrounding the soft tissues that were otherwise unremarkable without any bone involvement, fracture, or any other sign of aggressiveness in the form of bony destruction or periosteal reaction. Plain chest X-ray and abdominopelvic US were unremarkable. Local ultrasound examination showed a well-circumscribed echogenic mass splaying soft tissue planes. However, due to resource limitations, advanced cross-sectional imaging such as computed tomography (CT) scan or magnetic resonance imaging (MRI) was not performed.

Regional lymph node biopsy specimens from nearby inguinal lymph nodes showed reactive changes and no histological evidence of malignancy or granulomatous reactions, a chest X-ray was performed to rule out pulmonary metastases. This effectively excluded any suspicion of metastatic disease or other malignant causes of the swelling.

A punch biopsy from the calcified mass was carried out. Histopathological features typical for idiopathic tumoral calcinosis showed lobulated deposition of amorphous calcium with cystic space surrounded by foreign body giant cell reaction with chronic inflammatory cells without any features of malignancy or inflammatory reaction.

**Figure 2:**
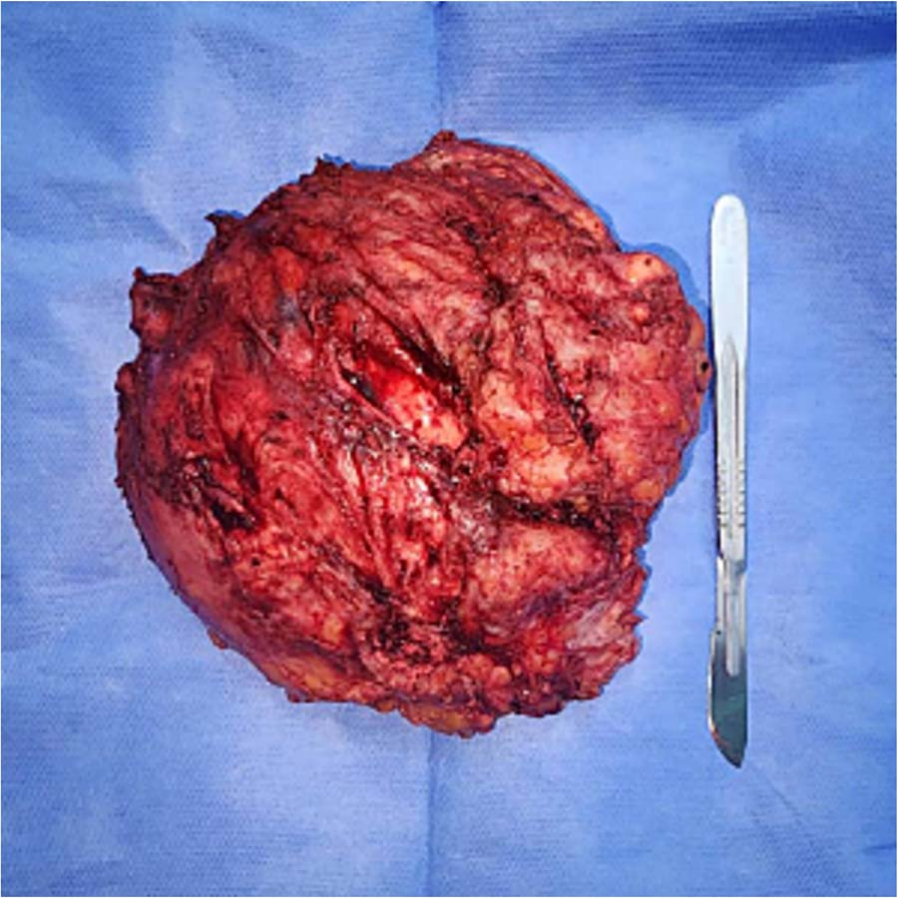
Shows the excised mass measuring 16 × 12 cm.

**Figure 3:**
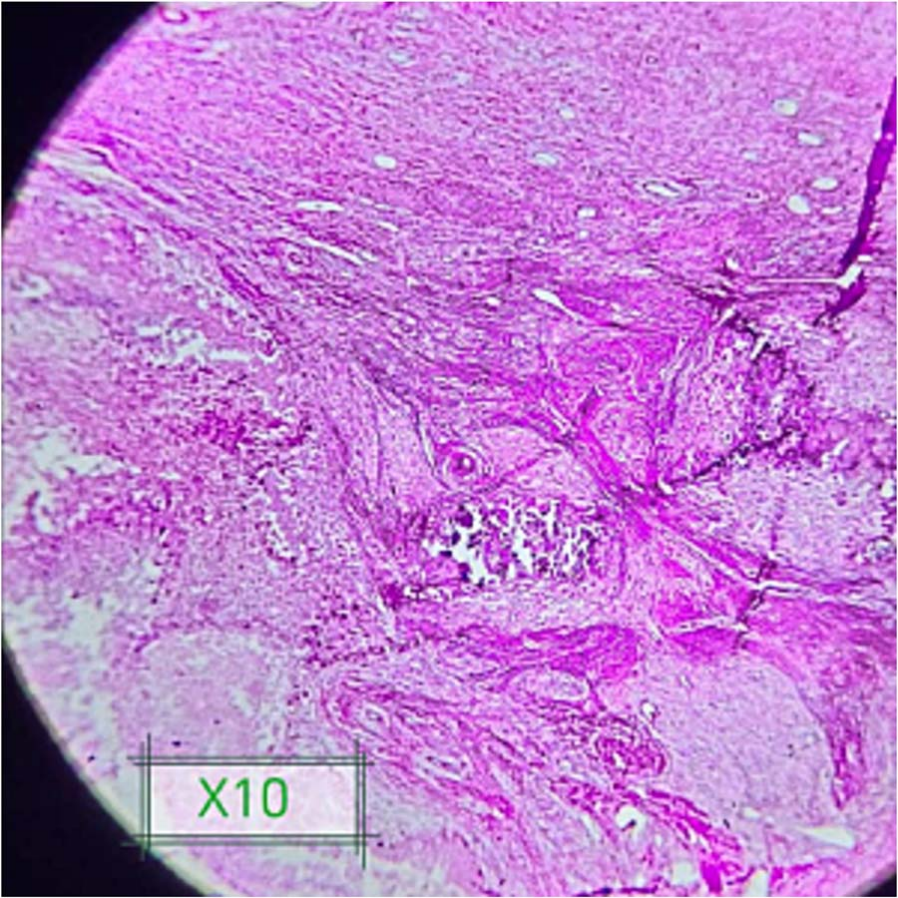
Shows foci of amorphous calcium deposits surrounded by foreign giant cells and fibrosis.

The patient underwent wide surgical excision of the mass under spinal anesthesia; it was completely removed without complications (Fig. 2). The histopathological results from the excised tissue showed features of idiopathic tumoral calcinosis with large, calcified deposits encapsulated by fibrous tissue, without features of malignancy or infection (Fig. 3).

The patient was discharged on the second postoperative day. The surgical drain was removed on day 10 and sutures at 2 weeks. The wound has remained clean without any complications.

## Discussion

Idiopathic tumoral calcinosis is a very rare benign condition due to deposition of calcium phosphate crystals into soft tissues, particularly in the periarticular region. The present case report describes a rather unusual presentation of idiopathic tumoral calcinosis in the left thigh of a 38-year-old Sudanese female and thus represents the first case to be reported in the literature in Sudan. In addition, this case report is intended to raise awareness for this uncommon clinical entity in geographical locations where it has never been reported before.

### Clinical and diagnostic challenges

Given its variable presentation, idiopathic tumoral calcinosis is often presented late. Our case was a painless, progressive swelling developing insidiously over a period of 1 year, which is also true according to descriptions of the condition in the literature [1–3]. The final diagnosis in the absence of any systemic metabolic disturbance like hyperparathyroidism or chronic renal failure was possible only after advanced imaging and histopathology.

Radiologically, the features in this case confirmed the previously described features of idiopathic tumoral calcinosis, including the characteristic “chicken wire” pattern of calcification on X-ray and well-circumscribed echogenic mass on ultrasound [7]. This characteristic radiological finding is useful for distinguishing tumoral calcinosis from other calcifying lesions, such as myositis ossificans and calcified hematoma. Also, the absence of associated bony erosion or periostitis excluded more aggressive or malignant conditions [5, 8]. However, due to resource limitations, advanced cross-sectional imaging such as CT or MRI, which could have provided further details on soft tissue involvement and deeper structural relationships, was not performed.

Histopathological examination remains the gold standard for definitive diagnosis. In this case, the biopsy showed large, calcified deposits encapsulated by fibrous tissue with no evidence of malignancy or inflammatory reaction. These findings go hand in glove with other reports in the literature, such as those by Laasri *et al.* and Jakka *et al.*, which describe the process of idiopathic tumoral calcinosis as a non-inflammatory one [1, 6].

### Management and outcome

Surgical excision, besides other therapeutic options, stands as the cornerstone of symptomatic or functionally impairing lesions of idiopathic tumoral calcinosis. In our case, the mass was excised completely under spinal anesthesia without any complication. The postoperative course of the patient was uneventful, and she remained asymptomatic for 6 months, up to date, of follow-up, consistent with favorable outcomes reported by other series of studies [3, 4].

Despite successful surgical intervention, recurrence is a cause for concern in idiopathic tumoral calcinosis. Although not studied, the recurrence is related to incomplete excision and underlying metabolic anomalies, neither of which applies to this case [2].

### Geographical and epidemiological considerations

Idiopathic tumoral calcinosis has geographical predilection, and most cases have been described among people of African and Middle Eastern origin; most of the cases were diagnosed during childhood or early adulthood [5, 9]. Thus, this case extends the demographic and geographic scope to include an adult female from Sudan. A few reports from countries like India, the Philippines, and Korea have also reported similar cases, drawing attention to the fact that this rare condition indeed has worldwide distribution [5, 8, 9].

## Conclusions

This case will justify the consideration of idiopathic tumoral calcinosis in the differential diagnosis of calcified soft tissue masses while considering underreporting from various parts of the world. Diagnosis and management require the assistance of different diagnostic modalities in the form of imaging and histopathology. Further studies regarding pathophysiology, epidemiology, and long-term outcomes are warranted in this infrequent condition, especially in underserved populations.

## Research registration

Not done (no new surgical technique or new equipment/technology used).
